# Observations on the Puerperal Fevers Which Occurred at the Maternité of Paris in 1829; on the Various Modes of Treatment That Were Adopted, and Particularly on Mercurials, Emetics, and Bloodletting

**Published:** 1830-10

**Authors:** M. Tonnelle


### PUERPERAL FEVERS.

### *Observations on the Puerperal Fevers which occurred at the Maternité of Paris in* 1829*; on the various Modes of Treatment that were adopted, and particularly on Mercurials, Emetics, and Bloodletting.* By M. TONNELLE.*

IN the preceding articles which we have given upon this subject, M. Tonnellé describes simple inflammation of the uterus and its appendages, and suppuration of the veins and lymphatics. We now arrive at his third division of the morbid appearances of the uterus, which consists of *a softened or putrescent state of the uterus*. This morbid alteration had been long overlooked; but lately it has been the theme of many investigations, particularly with the German pathologists, who have much improved our knowledge of the subject. Very recently, LUROTH of Strasbourg, and Dr. DANYAN, have given very correct descriptions of it.

A softened condition of the uterus, after having been frequently observed in the first half of the year 1829, and especially in January, disappeared almost entirely during the months of July and August, when uterine phlebitis was very frequent. In September and October it was again common; during November and December it disappeared, and at this latter period the mortality among the puerperal patients was very trifling. It existed in two principal forms, of which Boër and Luroth have made two distinct diseases: "*Ramollissement,*" properly so called, and a putrid condition, which are evidently but different degrees of one affection, as M. DESORMEAUX has observed.† In the first form, the internal surface of the uterus alone was softened. It presented itself in superficial patches, of a reddish brown colour, and of irregular form, which occupied various points of the surface. The limits of these patches were not strongly marked, the diseased tissue being confounded with the healthy structure by insensible shades and gradations. In the second variety, the softening was deeper, and of greater superficial extent. It sometimes implicated the whole substance of the body or neck of the uterus; and then the tissue of the organ was so soft, that it was penetrated when taken hold of by the fingers. In both cases the softened parts had a strong putrid odour; but we cannot affirm that this odour ema-

* Archives Gen. Avril 1830.

† Diction. de Méd.

nated from the altered tissue. It appeared frequently to arise from the putrescent membrane which almost constantly lines the internal surface of the uterus in this case, and which is observed in many other conditions. The superficial softening of the uterus was usually combined with some other morbid alteration, as inflammation of the uterus, peritonitis, or uterine phlebitis, and it has not appeared that its existence had much influence upon the progress of the symptoms. The second variety was also sometimes united with other disorders, but it generally constituted the principal alteration, and frequently the only one, and it constantly gave to the disease a well-marked typhoid character, the progress of which was most rapid. The following example, and many of those recorded by Danyan, establish this fact.

Case XV. *Putrid State of the Uterus, accompanied by severe Symptoms. Death in twenty-four hours.*

Roy. . ., æt. thirty, of a good constitution, was delivered after a favorable labour on the 10th October. In the night of the 12th she had some pain in the abdomen, accompanied by shivering. In the morning the belly was swollen and tender to the touch; features drawn; tongue dry; pulse small and frequent. Instead of the lochia, there was a fetid puriform discharge. In the day the patient was delirious and much agitated. In the evening she fell into a profound stupor, became cold, and died twenty-four hours from the attack.

Leeches were first applied to the belly, and mercurial frictions were freely employed.

*Dissection.* The uterus occupied nearly the whole cavity of the pelvis. About two thirds of its body, and the whole of its substance, were reduced into a brown pulp, which retained no traces of organization, and broke easily under the finger. Its internal surface was covered with a thick layer of semi-fluid matter, brown and very fetid. In the peritoneal cavity was a small quantity of bloody serum. All the organs were soft and flaccid.

Some of the septic matter, absorbed during the examination of the body, from an excoriation on the finger, almost immediately produced severe local and general symptoms; and still these deleterious properties could hardly be the result of putrefaction, for death had only taken place eighteen hours before, and the body had not undergone the slightest decomposition.

What is the nature of this alteration of the uterus? Is it a real gangrenous degeneration? or is it, on the contrary, a simple softening, analogous to that we observe in other organs, as the brain, heart, stomach, &c.? Are we to consider inflammation as the cause of it? The brown colour of the softened parts, the fetid odour which they exhale, and the softened condition itself, are evidently the only foundations upon which the idea of gangrene can rest. But it may be doubted whether the existence of these characters is sufficient to justify the opinion. The brown colour is not always the constant, nor particularly the immediate, effect of gangrene: it frequently exists in many other alterations, quite different from the preceding; as in a softened state of the stomach, œsophagus, &c., and the infiltration of a certain quantity of blood in the texture of the tissues is sufficient to produce it. The ramollissement, in our opinion, rather bears evidence against a gangrenous degeneration, than in favor of its existence. In the deep-seated gangrenes which affect the muscular substance, and which may be compared to that which attacks the stomach, the mortified tissues are indeed flaccid and shrunken, but they are not so soft as to be penetrated by the finger. As to the odour, we have already observed that it appears to arise less from the degenerated parts, than from the putrid matter which adheres to the surface of the uterus.

Another more decisive fact bears upon this question. The morbid alteration of which we are now speaking diminishes progressively, and ceases insensibly. Gangrene, on the contrary, is always limited and circumscribed. Different degrees of softening may be conceived, but not of mortification. We are inclined to believe that this alteration is nothing more than a simple ramollissement. But what share has inflammatory action in its production? Pfeuffer, Wenzel, and Danyan, attribute it to inflammation. Professor Boer, Jœrg,* and most of the German writers, according to M. Luroth, are of a contrary opinion, and each supports his view by plausible arguments. The important question, however, to ascertain is, whether inflammation is accessory and secondary, or whether it is essential and primary. It is easy to solve this question when it is thus stated. Ordinary inflammation, however severe it may be, presents during its developement several successive and re-

* M. Tonnellé gives no reference; but we find that Jærg is decidedly of opinion that this putrescent state of the uterus does not result from inflammatory action. Jœrg *Handbuch der Krankheiten des Weibes,* par. 708 et seq.—Ed.

gular stages, and certain constant and determinate symptoms, which are not found in the case we are now considering. It never, from its beginning, produces the very severe symptoms which almost always accompany ramollissement of the uterus. It does not destroy life with such terrible rapidity, especially when it affects an organ which is not immediately necessary to the existence of the individual. It appears evident, then, that inflammation in such cases is but an accessory phenomenon, and a sort of veil behind which is concealed the really active cause. It is difficult to determine the precise nature of this cause: we have many reasons for believing that it depends upon a vitiated condition of the blood, but we confess that facts are yet wanting to support this, at present, hypothetical opinion.

Such are the various morbid appearances which we have observed in puerperal fever; a disease which is as complicated in its structural lesions, as we shall shortly prove it to be in its symptoms. The following tables present a general view of these different alterations.

In 222 bodies of patients who had died of puerperal fever, we found peritonitis in 193; disease of the uterus and its appendages in 197; there were various combinations of morbid appearances of the uterus and peritoneum in 165 cases; in 28 the peritoneum alone was affected; in 29 the uterus alone. If we now state separately each alteration of the uterus, as we have done in our description, we find, 1st. *Alteration of the body of the uterus:* simple metritis, 79; superficial softening, 29; softening of the substance of the organ, 20; ovarial inflammation, 58; ovarial inflammation with abscess, 4. 2d. *Alteration of the uterine vessels:* pus in the veins, 90; pus in the lymphatics, 32; pus in veins, lymphatics, and also in the thoracic duct, 3; pus in the vessels, with inflammation and suppuration of the lumbar and inguinal glands, &c. 9.

It will be perceived that the morbid appearances of the uterus, considered separately, were much more numerous than those of the peritoneum. Their number is, indeed, greater than that of the bodies examined; a result which may at first appear paradoxical, but which depends upon several different alterations of the uterus, &c. having been detected in the same subject. Let us now consider the most important of these combinations. 1st. *Suppuration of the veins* was accompanied by a similar state of the uterus in 32 instances; by putrescence, or ramollissement, in 11; by metritis and ramollissement together, in 5; by peritonitis alone, in 34. In eight cases there was suppuration in the veins, with no other morbid appearance. 2d. *Suppuration of the lymphatics,* with that of the veins, in 20; with suppuration of the uterus, in 13; with ramollissement, but without suppuration of the uterus, in 6; with simple peritonitis, in 3; with no other alteration, in 2. 3d. *Inflammation of the ovaria,* with simple peritonitis, in 29; with various alterations of the uterus, in 27; with simple metritis, in 8; with ramollissement, in 7; with suppuration of the vessels, in 12; with a combination of all these morbid alterations, in 16.

It is a remarkable fact, that in 134 cases the veins or lymphatics of the uterus contained pus. Whether this secretion was formed in the vessels, or carried into them by absorption, is a very secondary question. The effects arising from such a vitiation of the blood are alone of importance, and they are the same in all cases. These effects are almost always fatal, and, to the danger of the disease, they add the still greater danger of a purulent infection of the whole mass of blood. The knowledge of these facts must be valuable to us: many errors will now be corrected, and many difficulties explained; and, indeed, a new character altogether is given to puerperal fevers. The morbid appearances in the lymphatic system, in the glands, and even the thoracic duct, which we so frequently found, have not been before mentioned, even by the most modern observers. In thirty-two cases of suppuration of the uterus, the veins contained pus, and it might be presumed that this secretion was carried into the vessels by absorption. But, on the other hand, the same appearance existed in eleven cases of ramollissement of the uterus without suppuration, in thirty-four cases of simple peritonitis, and in eight subjects there was no *other* morbid alteration whatever. It is difficult to conceive that the pus which was detected in the vessels was absorbed from the peritoneum, because there is no direct communication between these parts; and, if this were the case, absorption of matter would have taken place by other veins also; besides, the effusion produced by inflammation of the peritoneum is generally serous, and very different from the pus contained in the vessels of the uterus. We must at least admit, then, that, in more than one half of the cases, the pus was primarily formed within the veins: we doubt whether this was the fact in the other instances.

From the tables just given, it is clear that the terms *peritonitis* or *metro-peritonitis,* however applicable they may be to particular cases, can never be taken as the general designation of that species of disease of which we are now treating. Sometimes the term *metro-peritonitis* would be too comprehensive, at others too restricted in its meaning; and, besides, it involves in one common idea various and essentially different forms of disease, and leaves unnoticed the humoral element which we have shown to play so important a part in the malady, and also the nervous, the incontestible influence of which will be afterwards shown. It is true that the term puerperal fever does not embrace all these ideas, but it presupposes nothing, it fixes nothing, and therefore applies equally to all the forms of the disease. It should be retained as the general designation for the disease, and to particular cases the various denominations of peritonitis, metro-peritonitis, uterine phlebitis, &c. must be applied. Independently of the various morbid appearances already described in these cases of puerperal fever, there were others which were secondary or accidental, that deserve mention.

The thoracic and abdominal viscera were variously diseased; in fourteen cases there were abscesses in a great number of muscles; in three, depositions of blood; in two cases abscesses were found in the hip-joint, in two in the elbow-joint, and in six in the knee-joint. To these various lesions others were frequently joined, less susceptible of precise description. In many cases all the organs were found gorged with blood, the lungs, the liver, heart, brain, &c.; or these parts were softened, flaccid, or withered, as it were. The intestinal tube was usually distended by gaseous secretions. The mucous membrane almost always healthy, and of a perfectly white colour, was sometimes covered with an enormous quantity of bile mingled with mucus, or with a brown, thick, fetid matter. The bladder was often inflamed. Lacerations and superficial, or deep, sloughs of the perineum were frequently observed. The blood was sometimes rich, coagulated, and of a fine red colour; at others liquid, of a rose-like hue, or of a very dull appearance. No other lesion was ever observed in the brain than an injected or softened state, which was also found in other organs.

*Of the symptoms of puerperal fevers.* Three principal varieties of the disease were observed: the inflammatory, typhoid, and an anomalous or ataxic form. The inflammatory form is either well marked and durable, or, on the contrary, ephemeral and transitory. In the first case the disease is ordinarily connected with simple inflammation of the peritoneum or uterus; and, among the secondary alterations above mentioned, vascular engorgement of the viscera, pleurisy, pneumonia, pulmonary apoplexy, hypertrophy of the heart, hydrothorax, or hydrocardia, were those which were most frequently added. The second kind usually appeared at the beginning of those cases in which pus was found in the vessels, and sometimes during the first period of ramollissement. It mostly lasted for several days,* and then disappeared suddenly, giving place to another order of symptoms, which will be presently described. Sometimes this species of attack was so fugitive that it scarcely extended beyond a few hours or a single day.

Inflammatory puerperal fevers present two well-marked periods; the first of vascular congestion, the other of suppuration. The former is generally announced on the second or third day from delivery by rigors, and pain sometimes confined to one part of the abdomen, the hypogastrium, or iliac fossa, and sometimes extending over the whole of these regions. To these incipient symptoms are soon added a suppression or diminution of the lochial discharge, shrinking of the breasts, nausea, vomiting, general heat, hard and quick pulse; the face is alternately flushed and pale; eyes brilliant and injected; head heavy and painful; tongue dry and red, or covered with mucus; great thirst. Secondary vascular congestions almost always take place; and as the head, lungs, skin, alimentary canal, or liver is thus affected, either symptoms approaching to delirium are observed, or dyspnœa with feeble breathing, or abundant sweats, or enormous secretions of mucus and bile. These sanguineous congestions were sometimes so sudden as to produce apoplexy of the lungs or liver, or extravasation of blood into the pleura or pericardium. Most of these secondary phenomena greatly increased the severity of the disease, but this was not always the case when sweats and free alvine evacuations occurred. From the suspension of these evacuations, the local pain was frequently exasperated, and at their return much ameliorated. This remark was made by many of the ancient observers, and particularly Van Swieten. M. Desormeaux, so far from endeavouring to check these diarrhœas, was frequently anxious to provoke them. May not such evacuations cause a general weakening of the system, and tend powerfully to unload the abdominal vessels, and thus as efficaciously lead to the solution of the disease as strict

* The term "*ephémère*" is not, then, very correctly applied.—Ed.

diet and bleeding.* It is in this manner that such evacuations have appeared to us to be evidently critical phenomena.

The duration of the first period of the disease is so variable, that it is hardly possible to fix its limits: this difference depends less upon idiosyncrasies and individual predispositions than upon the constitution of the atmosphere. In sporadic cases, indeed, a certain regularity was observed; but in the epidemic type there was nothing fixed nor precise. The transition from the first to the second period was frequently accompanied by a temporary amendment and renewed rigors of short duration, and it is doubtless this circumstance which has induced some physicians to class the disease among remittent fevers. When effusion or suppuration was formed, the pain sometimes became dull and deep-seated; but it often retained its acute character, and even became more severe. At the same time the abdominal parietes were distended by flatus, which also pushed up the diaphragm, and thus added greatly to the already existing dyspnœa. It was not uncommon at this period for a fetid discharge to take place from the vagina, and pus to form in the breasts. To the vascular congestions observed in the first stage, local inflammations of the pleura or lungs, or copious evacuations of bile, frequently succeeded, and thus a serious complication of disease was formed. Sometimes the agitation and general heat continued to the end of the disease, the pulse preserving its rapidity and hardness, the strength at the same time not flagging, and the patient dying in full possession of her faculties. At other times, on the contrary, all the functions were oppressed, and the fatal termination occurred after long suffering. When the disease was about to end favorably, abundant sweats usually broke out: this phenomenon has been noticed by most observers, and particularly Hoffman (Medicina Ration. t. iv. p. 317,) Leake (on Childbed Fever, p. 56,) and Doublet, who sometimes observed these sweats continue for twenty-four, thirty-six, or even seventy-five hours. Diarrhœa also almost always occurred at this period.

*The typhoid form* was most frequent, and existed in most cases of ramollissement of the uterus, or suppuration of

* Our own experience leads us to answer this question in the negative. In inflammatory puerperal fever, active purgatives are often of great service, but free spontaneous diarrhœa has not appeared to us to exert a similar influence upon the disease. We would not endeavour suddenly to check such evacuations, but we should look upon their continuance with alarm.—Ed.

the veins or lymphatics, whether the pus was primarily formed in the vessels or carried into them by absorption. It was generally preceded by the ephemeral inflammatory form above described. It may perhaps be objected, if typhoid puerperal fever is mostly the result of local inflammation, that a distinct species should be made of it. We reply, that in most cases the typhoid form *is* a secondary effect, but *an effect* which in its turn becomes a *cause,* and a cause so powerful as to impart a new character to the disease, and to change its nature, and thus to create special therapeutic indications. The remote causes of typhoid puerperal fever almost always may be referred to suppuration of the veins or lymphatics of the uterus, and to ramollissement of this organ. The immediate cause is an alteration of the blood, which is sometimes of an unknown nature, and insusceptible of demonstration, as in cases of ramollissement. It is sometimes evident and palpable, as in suppuration* of the vessels. To these primitive morbid alterations were ordinarily added several others, as abscesses of the lungs, liver, pancreas, muscles, joints, pleurisies, circumscribed pneumonia, gangrene of the lungs, disorganization and perforation of the stomach, flaccidity of the tissues, and frequently ramollissement of all the organs, a fluid and altered aspect of the blood, &c. Some of the symptoms were the immediate result of purulent absorption; others, the secondary effect of different organic lesions, consecutive to infection of the blood. From the commencement there was an air of languor and depression, either with or without diminution of the abdominal pains, which never deceived the well-practised eye of M. Desormeaux. Later in the disease, the intellectual faculties were disturbed, skin of a yellowish tinge, the face sometimes of a purplish blush, an appearance of prostration, eye dry and dull, buzzing in the ear, impediment of speech, mild and gloomy delirium, stupor, and but rarely great agitation and shrieks. To these symptoms were constantly added a very quick, small, and irregular pulse, dyspnœa, swelling of the abdomen, local sloughs, viscid sweats, sanious discharge from the vagina; brown, fetid, and very abundant alvine evacuations passed involuntarily, and very different from those observed in the inflammatory form. These symptoms were not all present, excepting in a few of the

* We again remind the reader that M. Tonnellé implies by the term "*suppuration des Vaisseaux,*" nothing more than the simple fact of *pus* being found in the vessels.—Ed.

severest cases; but some of these essential features were always observed, as may be seen by referring to the cases before detailed.

In the midst of this great disorder of the functions, the secondary alterations of the solids were sometimes not detected. Their developement was generally very rapid, and the symptoms insidious and obscure, and so confounded with those of the principal disease that they escaped the most scrupulous attention. The local diseases which were most completely latent were those of the liver, pancreas, and joints; certain cases of pneumonia and pleurisy; others, as gangrene of the lungs (Case vii.), and disorganization and perforation of the stomach (Cases vi. xiv.), sometimes gave rise to symptoms which led to a suspicion of the nature of the mischief during life, but which were never sufficiently well marked to allow the practitioner to detect the beginning of the local disease, or to follow its progress. Abscesses in the muscles commenced insidiously, and might easily have escaped a superficial examination; but M. Desormeaux never failed to detect them, by the conscientious attention he bestowed upon his patients. When the disease terminated favorably, the matter in these abscesses was sometimes gradually absorbed, and no trace was left of its existence, or it gradually approached the surface, and its evacuation became necessary. When the abscess was of small extent, the parts were easily restored to a healthy state; but otherwise, extensive depositions of pus followed, with deep-seated destruction of the tissues, and the life of the patient was thus exposed to new perils.

The *anomalous* or *ataxic* form was rarely observed except during the course of an epidemic. Its chief signs were, great irregularity in the march of the symptoms; agitation, delirium alternating with depression, syncope, paroxysms of suffocation, irregularity in the circulation, and heat of the body; and frequently marks of intense peritoneal or uterine inflammation. Upon dissection, there was scarcely any appreciable alteration detected, and certainly none which could explain the death of the patient. To give a more correct idea of this variety of the disease, we detail a few cases.

Case I. ***Puerperal Fever, with Ataxic Symptoms from the commencement.***

Bartholo. . ., æt. twenty-one, of good constitution, had a natural labour, August 29th. Second day from delivery, she had prolonged rigors, followed by free sweating and some abdominal pains. Evening: Great agitation and violent delirium, which lasted the greater part of the night. Morning: Calm, but somewhat depressed; abdomen tender upon pressure; liquid stools, and very frequent; breasts empty and flaccid; pulse small and quick; lochia continue.

Fourth day. Syncope and several bilious vomitings; tumefaction of the belly; pains had ceased. Evening: A violent attack of dyspnœa came on, and gave place to profound prostration.

Fifth day. Abdominal pains returned, together with agitation and delirium. Diarrhœa ceased spontaneously.

Sixth day. Cold sweats, quick breathing, and irregular pulse; vomiting and involuntary alvine discharges. Died the next day.

Fifty leeches were twice applied, and afterwards mercurial frictions were employed, in the quantity of two ounces each day. A few doses of calomel were given. M. Desormeaux superintended the treatment.

*Dissection, twenty-four hours post mortem.* A small quantity of limpid serous fluid in the abdomen, and a slightly injected appearance of the peritoneum around the uterus. Two or three of the uterine veins contained a small quantity of turbid serum, which appeared to be the rudiment of pus; uterus contracted and healthy. The mucous membrane of the stomach and intestines quite white throughout its whole extent. Lungs engorged, but crepitant. Heart half filled with blood of a brown colour. Brain firm, and slightly injected. Other organs natural in appearance.

Case II. ***Puerperal Fever, with Ataxic Symptoms, consecutive to Inflammatory Symptoms: Progress acute.***

Lina Hof..., æt. twenty-two, weak and nervous, miscarried the first month of her pregnancy, from which she had suffered much: pains in the loins and hypogastrium, and rigors, almost immediately followed abortion. Evening: Severity of symptoms increased; abundant bilious vomiting.

Second day. Great prostration; taciturn delirium. Pressure upon the abdomen appeared painful, from the expression of the countenance. Lochial discharge uninterrupted. During the day she remained in a state of profound coma; but in the evening she was restless and agitated, and frequently endeavoured to get up. Repeated vomiting.

Third day. Recovery of consciousness, and vague and confused expressions of complaint; but the eye was dull, extremities cold, and pulse irregular. Death.

The inflammatory symptoms from the commencement were treated by ipecacuanha;* forty leeches were then applied to the abdomen, and afterwards free mercurial frictions, blisters, and sinapisms were used.

*Dissection, thirty hours post mortem.* Peritoneum slightly injected, and in the abdomen were about four ounces of rose-coloured fluid. Internal membrane of the uterus healthy, and covered with vermilion blood: towards the cervix was seen a layer of concrete pus. A semitransparent lymph filled some of the uterine veins; most of the others were empty. Other parts healthy; the brain was carefully examined.

Case III. *Puerperal Fever, with prolonged Ataxic Symptoms, alternating with inflammatory Symptoms.*

Virginie Car..., æt. twenty-one, of weak and nervous constitution, was favorably delivered at the eighth month of pregnancy. At first no particular symptom arose; but on the fourth day she had shivering and a long fainting fit.

Evening, and next day. Great pains in the abdomen, with diarrhœa and fever. Fifty leeches were twice applied.

Sixth day. Attack of dyspnœa, which was relieved by thirty leeches to the side.

Seventh day. Free from suffering: diarrhœa has ceased.

Eighth. Syncope, and some pains in the abdomen.

Ninth. Another severe attack of dyspnœa, for which a blister was applied to the chest.

Tenth. In the morning free from complaint, but in the evening delirium occurred.

The following day, coma, which was soon followed by death.

The lochial discharge continued throughout the course of the disease, at first sanguineous, and then serous, and in diminished quantity.

Upon dissection, the peritoneum was found slightly injected, and it contained about a pint of rose-coloured serum. Uterus of large size: its tissue white and firm, and its veins half filled with fluid blood. Lymphatics natural. The internal surface of the uterus was covered with brown fetid blood, but in other respects it appeared healthy. Cervix uteri had an appearance of ecchymosis, and was

* Doses not mentioned.—Ed.

covered with a thin greyish exudation. Other organs healthy.

These examples are sufficient to give an idea of the ataxic form of puerperal fever. It will be observed that the morbid appearances in these cases were neither numerous nor important, and it would be difficult to connect them with the symptoms which existed during life. The lesions which were detected may, indeed, to a certain extent, explain the pains, vomiting, and fever, which occurred, although these symptoms usually correspond with more extensive and serious alteration of parts. But to what organic lesion can we refer the agitation, delirium, prostration, syncope, cold sweats, dyspnœa, rapid and small pulse, and the irregular paroxysms and unequal progress of the disease. What was the nature and *point de départ* of these symptoms? In the first place, it is evident that they were such as the ancient writers denominated "*ataxic*," and, symptomatically speaking, the designation was happily selected, for disorder and irregularity are its distinctive attributes. As they were ignorant of the *point de départ*, they could do no more than name them by one of their most striking characteristics. But now that the progress of medicine permits us to pass from the observation of symptoms (to which the ancients were greatly attached,) to the study of the various organic lesions which correspond with them: now, when we can no longer conceive the existence of one without the other, the expression of "ataxic symptoms," or the abstract term of "ataxia," which comprehends them all, will not alone impart to us the idea of a peculiar kind of functional derangement, but also of some organic alteration from which the symptoms originate. But what organ is diseased in these cases? It cannot be doubted that ataxic symptoms in general, as well as those particular ones we have just enumerated, emanate from the nervous system. But in what manner is this system altered? Here arises the difficulty; for we may assert what it is not, but not what it is. We know that it is not always a lesion of that kind called *irritation** or *inflammation*, which leaves after death appreciable traces of their existence; and that, consequently, whatever may have been said to the contrary, the term "ataxia" is not synonimous with cerebritis, encephalitis, as is proved by the previous cases. But it is as impossible, in the present

* It is to be regretted that M. Tonnelle has not stated what he conceives *are* the post-mortem traces of *irritation*.—Ed.

state of our knowledge, to determine the precise nature of this alteration, however well we may be acquainted with its effects, as it would be to show the cause of various other nervous diseases, as mania, epilepsy, chorea, &c. The term ataxia may therefore be retained without inconvenience: it ought, indeed, to be retained so long as we cannot ascertain the kind of lesion of the brain upon which ataxic symptoms depend. As we know not the essence of the disease, we could but substitute one term for another, without at all enlightening the question.

Let us now examine what connexion there exists between this state of ataxia and the puerperal disease, and whether the lesion of the nervous system was the result of the alteration of the uterus and peritoneum which was observed, or whether the contrary was the fact; and, in either case, what influence the one produces upon the other. If we refer to the previous examples, we find that the symptoms of disease of the peritoneum and uterus constantly preceded the ataxic symptoms, and that thus we might infer the former to be the cause of the latter. On the other hand, it appears probable that the disturbance of the nervous system interfered with the progress of the primitive malady, and arrested its developement. We cannot otherwise understand why the morbid appearances should have been so limited and so slight, especially in the first and third cases, where death did not take place till the sixth and seventh day.

In reference to the second question, how the developement of the ataxic symptoms is connected with peritonitis or metritis, we might endeavour to explain the fact by the sympathetic influence of the peritoneum and uterus upon the brain. It might, in addition, be urged that pregnancy and delivery exalt the sensibility of the woman, increase the susceptibility of the brain, and thus dispose the organ to disease. But we should scarcely know how to defend ourselves if it were objected that the term *sympathy* merely expresses the fact of the co-existence of both morbid conditions, without explaining it in any manner, and that it is better to remain in ignorance than to employ indefinite words, to which a positive meaning may at last be attributed that they really do not possess. It is therefore wiser to limit ourselves to the proof of the fact, without endeavouring to explain it. We must anticipate one objection which may be made relative to the morbid condition of which we have been speaking. It may be said, that if these ataxic symptoms are but the result of inflammation of the peritoneum and uterus, they ought not to be separately discussed, any more than vomiting and diarrhœa, which are also frequent accompaniments of these affections. The objection is merely specious: for, although the lesion of the nervous system appears to us to be consecutive, it is a new affection, which, as we have already remarked respecting the typhoid form of puerperal fever, absorbs, as it were, the primary disease, produces peculiar symptoms, a special danger, and, as the preceding cases show, destroys by its own power, long before the primitive alteration of parts could have done so. We ask, therefore, whether these characteristics are not sufficient to distinguish this kind of disease from the preceding, and to admit of its being made a special genus.

We have now described the three forms under which puerperal fevers have been offered to our observation, and we append a synoptical table which combines them all in one view, and classes them according to their varieties. We by no means assert that puerperal fever will always be exhibited in either of these forms, and that it can assume no other. The observation of a day cannot limit the range of disease. At different periods very different forms of maladies exist, and years of investigation must be required to embrace the whole.

***Table of the different Forms of Puerperal Fevers which have been described.***

| | | |
|---|---|---|
| Puerperal Fever, or Disease. | Inflammatory form, or simple inflammation of different organs . . . . . | Peritoneum, Uterus and its appendages. |
| | Typhoid form, or from alteration of the blood | *Consecutive* to suppuration of the vessels.<br>Concomitant with a state of putrescence or ramollissement. |
| | Ataxic form, or from lesion of the nervous system. | |

